# Consistency of T2WI-FS/ASL fusion images in delineating the volume of nasopharyngeal carcinoma

**DOI:** 10.1038/srep18431

**Published:** 2015-12-16

**Authors:** Meng Lin, Xiaoduo Yu, Han Ouyang, Dehong Luo, Chunwu Zhou

**Affiliations:** 1 Department of Diagnostic Radiology, Cancer Institute & Hospital, Peking Union Medical College, Chinese Academy of Medical Sciences, Beijing, China

## Abstract

Tumor extent assessment of nasopharyngeal carcinoma (NPC) is critical for delineating the radiotherapeutic target region. We aimed to investigate the use of the fusion images of fat suppressed T2WI (T2WI-FS) with arterial spin labeling (ASL) in measuring the volume of NPC. Two observers measured the volume of 21 untreated NPC using T2WI-FS, T2WI-FS/ASL (with PLD = 1.0, 1.5 and 2.0 s) fusion images and enhanced T1WI separately. Correlation and consistency were used to compare 1) measurements using T2WI-FS/ASL and T2WI-FS alone, taking enhanced T1WI images as a benchmark; 2) measurements between observers. Significant correlations existed between different series (r: 0.896~0.973). Measurements from the two observers using T2WI-FS/ASL had relatively higher intra-class correlation (ICC) (0.980~0.997) and lower within-subject coefficients of variation (wsCV) (14.76%~22.96%) when compared to using T2WI-FS alone (ICC: 0.978, 0.951, wsCV: 21.61%, 24.21%), while the T2WI-FS/ASL 1.0 s exhibited the best performance. Remarkably high ICC value (0.981~0.996) and relatively low wsCV (9.95%~17.91%) were obtained for the two observers using same series. Compared to those obtained using T2WI-FS alone, measurements made using T2WI-FS/ASL were more consistent with those made using enhanced T1WI. The T2WI-FS/ASL fusion images has the potential to be an alternative to enhanced T1WI, when contrast administration can not be performed.

Nasopharyngeal carcinoma (NPC) is a type of malignant head and neck tumor commonly seen in Asian population, especially in South China^1^. Radiotherapy or concurrent chemoradiotherapy are the main curative treatment due to the sensitivity of NPC to radiation. Three dimensional intensity modulated radiotherapy (IMRT) is commonly used to locate radiation target for tumor and high risk region, while protecting adjacent healthy tissue to reduce the radiation side-effect.

NPC is usually derived from nasopharyngeal mucosa. Nasopharyngoscope and biopsy are commonly practiced to confirm the pathology of NPC, however neither of the two is able to accurately assess the tumor extents as tumors often invade the surrounding deep tissues along the submucosa. Therefore, imaging is usually needed for tumor staging and also to delineate the target region of radiotherapy. The radiotherapy target regions are composed of gross tumor volume (GTV), clinical target volume (CTV) and planning target volume (PTV), and the GTV is the key region based on which CTV and PTV can be defined. Magnetic resonance imaging (MRI) is the optimal modality for NPC diagnosis and staging due to its excellent morphological contrast^2^,^3^. Conventional MR examination on nasopharynx consists of non-enhanced T1- weighted imaging (T1WI), fat suppressed T2-weighted imaging (T2WI-FS), diffusion weighted imaging (DWI) and contrast enhanced T1WI. The T2WI is a useful non-contrast sequence with high SNR, while the contrast enhanced T1WI has the advantage over T2WI for NPC diagnosis and staging due to the improved contrast noise ratio between tumor and surrounding tissue (such as fat and muscle)^4^,^5^. Contrast enhanced T1WI is also the only MR examination recommended by national comprehensive cancer network (NCCN) for NPC.

Arterial spin labeling (ASL) is a noninvasive MRI technique that uses blood as an endogenous tracer to image the perfusion effects in tissue, avoiding the danger such as allergy and nephrogenic systemic fibrosis (NSF) caused by contrast agent^6^. For ASL, the contrast between tumor and surrounding tissues is attributed to the relatively high blood flow (BF) in tumor as compared to in surrounding tissues, and combination of ASL with T2WI-FS images would improve the morphological description of tumor as the ASL images are usually of low spatial resolution. This study compares the consistency of T2WI-FS and T2WI-FS/ASL fusion images in assessing the local tumor volumes of NPC, taking contrast enhanced T1WI as the benchmark, and it was aimed to investigate the value of the T2WI-FS/ASL on delineating the radiotherapy target.

## Results

### Correlation of measurements between different series

The volumes measured by two observers using enhanced T1WI and those using T2WI-FS, T2WI-FS/ASL (PLD of 1.0, 1.5, 2.0 s) fusion images showed significant correlation. It is seen that the correlation coefficients of T2WI-FS/ASL (0.921 ~ 0.973) were higher than those of T2WI-FS alone (0.896, 0.910) based on both observers; and the correlation coefficient of T2WI-FS/ASL 1.0 s from observer 1 was the highest (0.973) while that of T2WI-FS from observer 2 was the lowest (0.896) (Table 1).

### Consistency of measurements between different series

**Figure 1:**
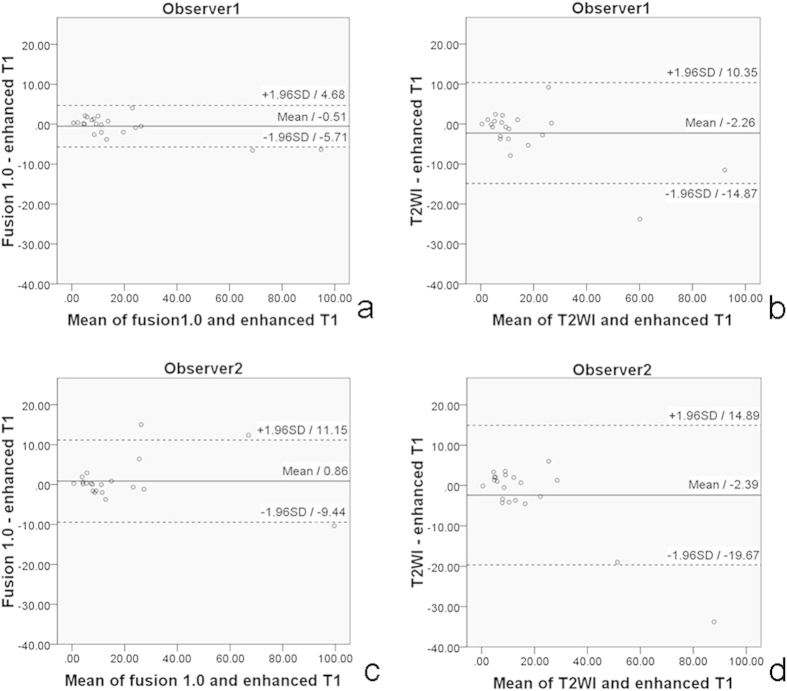
Bland–Altman plots together with 95% CI of NPC volume inter-series. Bland Altman plot of NPC volume measurements made at T2WI/ASL 1.0 s and enhanced T1WI by observer1 (**a**), at T2WI and enhanced T1WI by observer1 (**b**), at T2WI/ASL 1.0 s and enhanced T1WI by observer2 (**c**), at T2WI and enhanced T1WI by observer2 (**d**). Solid line and adjacent number indicates the mean difference, and whilst dashed line and number indicates the limits of agreement. The measurements of T2WI/ASL 1.0 s and enhanced T1WI by observer1 (**a**) showed fairly good agreement with the smallest SD among those four Bland–Altman plots.

**Figure 2:**
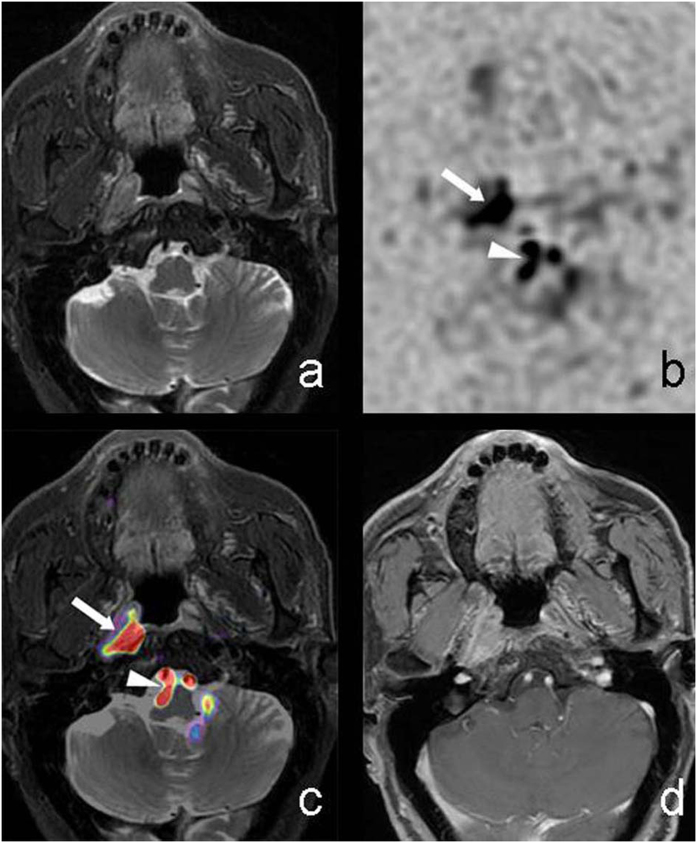
M71, Nasopharyngeal carcinoma invaded the right pharyngeal recess. T2WI-FS (**a**) showed poorly defined nodule in right pharyngeal recess with slightly higher signal intensity compared with surrounding muscle. CBF maps with reversed contrast (**b**) showed poor anatomical features and irregular nodular high signal intensity on the right region (solid arrow) and slightly back (arrow head) in the central region. T2WI-FS/ASL(with PLD = 1.0 s) fusion image (**c**) showed a clearly defined nodule in right pharyngeal recess covered with pseudo-color (solid arrow), and T2WI-FS showed flow void phenomenon of bilateral vertebral artery covered with pseudo-color (arrow head) that was not continuous due to the nasopharyngeal lesion. Enhanced T1WI (**d**) showed significantly enhanced tumor with similar extent to fusion imaging.

**Figure 3:**
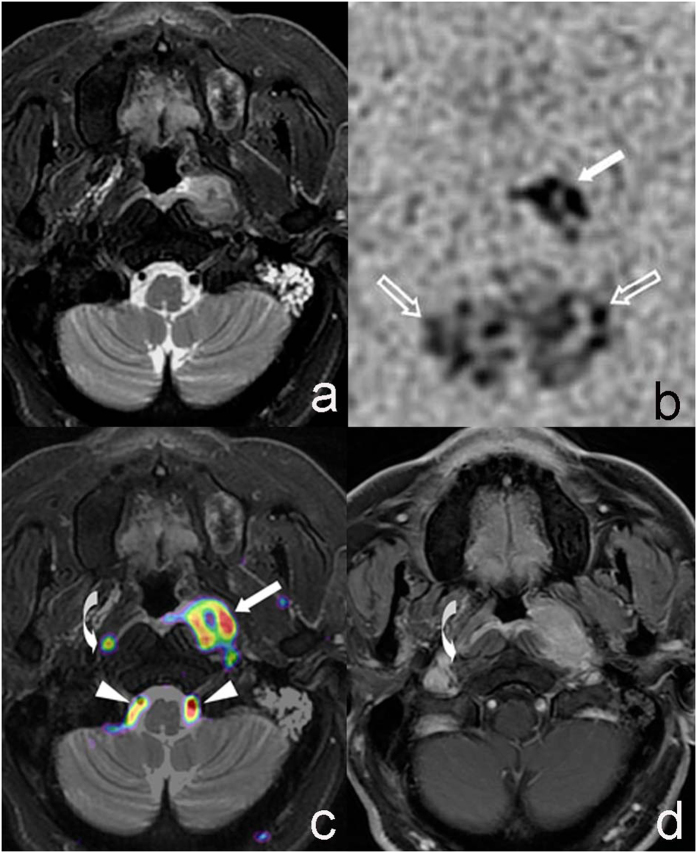
M51, Nasopharyngeal carcinoma invaded the left pharyngeal recess and parapharyngeal region. T2WI-FS (**a**) showed poorly defined tumor in left pharyngeal recess and parapharyngeal region with slight hyper-intense signal comparing to surrounding muscle. Thickened mucosa of parapharyngeal posterior wall near the tumor was observed. CBF maps with reversed contrast (**b**) showed nodular hyper-intense signal on the left (solid arrow) and patchy hyper-intense signal on the back (hollow arrow) region. T2WI-FS/ASL (PLD = 1.0 s) fusion image (**c**) showed well-defined tumor region invading left pharyngeal recess, space and posterior wall that was covered with pseudo-color (solid arrow), and T2WI-FS image showed flow void phenomenon of bilateral vertebral artery covered with pseudo-color (arrow head). A nodule with pseudo-color (curved arrow) was shown on the right retropharyngeal lymph node. Enhanced T1WI (**d**) showed contrast enhanced tumor with similar extent to fusion images and retropharyngeal lymph node (curved arrow).

**Figure 4:**
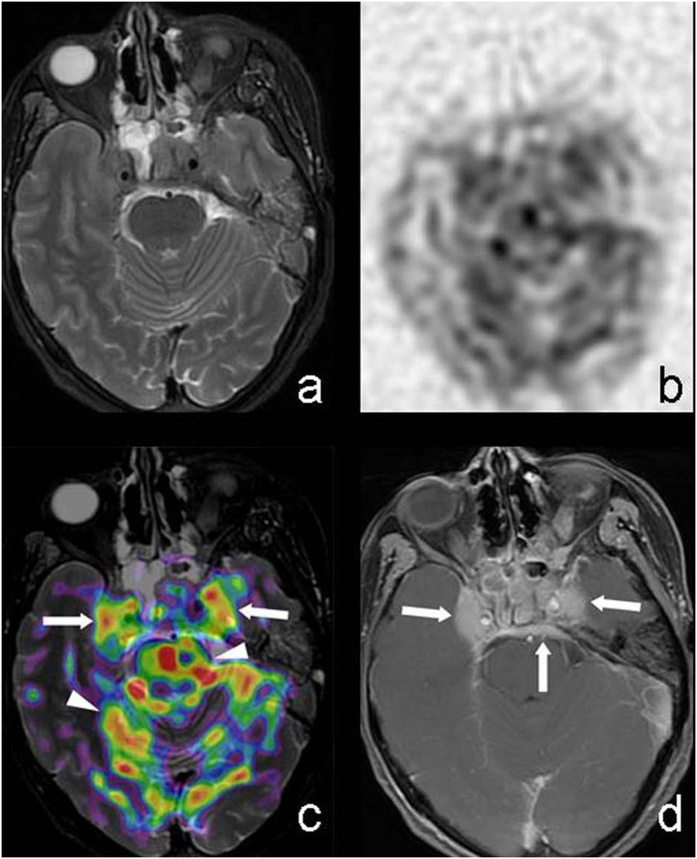
M39, Nasopharyngeal carcinoma invading sphenoid sinus, bilateral cavernous sinus and meninges. T2WI-FS (**a**) showed poorly defined tumor that had little contrast compared to surrounding tissue. CBF maps with reversed contrast (**b**) showed irregular hyper-intense signal area. T2WI-FS/ASL(PLD = 1.0 s) (**c**) showed tumor on sphenoid sinus, cavernous sinus covered with pseudo-color (solid arrow), which had clearer margin compared to the case of using T2WI-FS only. The patchy pseudo-color areas on brainstem and cerebellum (arrow head) which exhibited no lesion on T2WI-FS and discontinuous to nasopharyngeal tumor were considered to be normal high perfusion region. Enhanced T1WI (**d**) showed significantly enhanced tumor on sphenoid sinus, cavernous sinus and meninges (solid arrow).

Good agreement between inter-series measurements was received based on the Bland Altman analysis: lowest ICC (0.978, 0.951) and highest wsCV (21.61%, 24.21%) were obtained between T2WI-FS and enhanced T1WI; higher ICC (0.980 ~ 0.997) and relatively lower wsCV (14.76% ~ 22.96%) were obtained between T2WI-FS/ASL and enhanced T1WI. Among the three fusion series with different PLDs of ASL, the T2WI-FS/ASL 1.0 s exhibited the best performance with the highest correlation coefficient, highest ICC value and the lowest wsCV, and conversely the T2WI-FS alone (Table 2, Figs 1, 2, 3, 4).

### Consistency of measurements between two observers

Using the same series, remarkably high ICC value and relatively low wsCV were obtained between the two observers. The enhanced T1WI had the highest ICC value (0.996) and the lowest wsCV (9.95%), and similarly high ICC and relatively low wsCV were also obtained for T2WI-FS and three fusion images (Table 3).

### BF difference between NPC and lateral pterygoid muscle (LPM)

The measured BF of NPC were significantly higher than those of LPM on ASL with PLD = 1.0, 1.5 and 2.0 s with P values all less than 0.001 (Table 4).

## Discussion

Previous studies of DCE-MRI on NPC showed that perfusion images may assist in diagnosis and treatment in evaluating clinical stage, therapeutic effect, differentiating the tumor recurrence from post-therapeutic change, and predicting prognosis^7^,^8^,^9^. Enhanced T1WI MRI using intravenous contrast media (gadolinium) is the main series recommended by NCCN for NPC. Contrast media allows great image contrast between the tumor and surrounding tissue, which is much less obvious on non-contrast enhanced T1, T2 image. However, the contrast administration may have side-effects such as allergy and NSF^10^. Arterial spin labeling uses the endogenous blood as a contrast media to achieve perfusion imaging, and avoids the issues associated with contrast media administration.

Early studies on ASL were mainly focused on central nerve system, and then attention shifted to organ and tumor of neck and body in recent years. The study of Gillis *et al.*^11^ used ASL on the kidney of healthy volunteers and showed good agreement on measurements of kidney volume, whole kidney and renal cortical perfusion inter and intra MR scans. Christina Schraml ’s study on thyroid ASL^12^ found that the mean thyroid perfusion in patients with Graves disease (GD) (1596 mL/min/100 g) and Hashimoto thyroiditis (HT) (825 mL/min/100 g) were both higher than those of healthy control subjects (491 mL/min/100 g). Fujima N investigated the use of ASL on non-surgical head and neck tumor^13^, and concluded that pretreatment tumor BF (TBF) (121.4 mL/min/100 g) significantly decreased after treatment (24.9 mL/min/100 g), and post-treatment TBF was significantly higher in patients with residual tumors than those without.

This study using ASL on NPC have several advantages. Firstly, the spiral FSE based acquisition in ASL features high SNR as well as immunity to field inhomogeneity. Secondly, the nasopharyngeal image did not need motion correction as the fixed position had little motion artifacts. Our results showed that NPC demonstrated high perfusion in which BF was significantly higher than that of surrounding muscle. It indicated that ASL could help assessing the blood supply and distinguishing the tumor and surrounding normal tissue to define the tumor extent.

Previous studies of ASL were mainly focused on perfusion quantitative assessment, but less on morphological assessment. In a prostate ASL study, Cai W^14^, found high contrast-to-noise ratio (CNR) between cancerous and non-cancerous areas of the prostate (the highest to 13.7 ± 5.5). Therefore, ASL is able to provide good contrast between tumor and non-tumor regions, but the main issues of ASL are poor anatomical definition and lack of precise lesion location, as similar to DWI. Rosenkrantz AB^15^ combined DWI with T2WI-FS and found the fusion images could improve the efficacy of tumor detection remarkably. Our study conducted fusion of T2WI-FS with ASL images using different PLD times, in order to not only image the anatomical alternation but also the perfusion function difference of the tumor regions. Because the current benchmark for NPC staging and radiotherapy target delineation is contrast enhanced T1WI, this study measured the tumor volume on T2WI-FS, T2WI-FS/ASL fusion images and enhanced T1WI respectively, and assessed the consistency inter series and inter observers.

In this study, a 3D peudo-continuous ASL with FSE spiral readout was used. Compared to conventional ASL acquisition, this technique has three advantages: 1) high slice direction spatial resolution; 2) high SNR due to 3D encoding and peudo-continuous labeling; 3) FSE based spiral readout lead to minimal susceptibility distortion. These advantages realizes a good anatomical match with T2 images and help to improve T2WI-FS/ASL fusion images^16^. Observers found T2WI-FS/ASL fusion images provided more information than T2WI-FS alone and the tumor extents defined by fusion images were more consistent to those defined by enhanced T1WI. T2WI-FS alone is limited in defining the tumor extent as although NPC shows higher or slightly higher signal intensity than muscle, it has similar signal level to normal nasopharyngeal mucosa and cerebral cortex. T2WI alone does tend to under-estimate the tumor volume, consequently the volume measurement may be smaller than that of enhanced T1WI. In comparison, T2WI-FS/ASL fusion images feature additional perfusion information as compared to the conventional T2WI-FS, and would have increased diagnostic confidence of observers for determining the tumor extent (Figs 2 and 4). NPC and retropharyngeal lymph nodes showed highlighted signal intensity on ASL due to high BF, resulted in remarkable pseudo-color contrast in T2WI-FS/ASL fusion images; the muscle, bone displayed low signal intensity that is close to background noise on ASL due to low BF, resulted in little or no contrast on fusion image. Therefore, the fusion images were observed to define the tumor extent better than T2WI alone and provided tumor measurements closer to those from enhanced T1WI. Using the fusion images, the volumes measured were smaller by observer 1 and larger by observer 2, as compared to the enhanced T1WI, without a tendency to be under- or over- estimated. Other tissues with hyper or slight hyper intense signal on ASL map due to high perfusion also need to be distinguished from tumor. For instance, the regional artery (internal carotid artery, basilar artery and vertebral artery) exhibit low signal intensity on T2WI-FS due to flow void phenomenon, but small vessels within the muscle gap and brain tissue (such as brain stem, and part of cerebral cortex and subcortical areas) would lead to patchy high BF on perfusion map. However these regions can be ruled out as they are not in proximity to tumor regions and there is also no abnormality observed on T2WI-FS image. The invasion to skull base is more challenging to assess due to the complex structure, and might be influenced by adjacent brain tissues which feature high signal intensity on perfusion map. More cases are needed for achieving this goal.

Post-labeling delay time (PLD) is the time period for the labeled blood to travel from the labeling plane to the imaged region, and is an important factor as it influences BF value, CNR, and hence the disease diagnosis^14^,^17^. Our results showed that the resulting BF of tumor and normal muscle varied with different PLDs. The BF of NPC (from 74.67 to 81.19 mL/min/100 g) and muscle (from 15.01 to 20.04 mL/min/100 g) increased considerably when the PLD was changed from 1.0 s to 1.5 s, but it only slightly increased in NPC (to 82.52 mL/min/100 g) and muscle (to 21.15 mL/min/100 g) when PLD was increased to 2.0 s. In this study, BF of tumor was significantly higher than surrounding tissue with all of the three PLDs, and the consistency of T2WI/ASL (PLD = 1.0 s) to enhanced T1WI was the highest among those fusion images with different PLDs.

There were some limitations in this study. Firstly the sample size was small and could not be further stratified by tumor size and clinical stage. Secondly, this study only investigated the whole volume of NPC, without specification to the invasion of every location such as nerve, skull bone. Thirdly, the tumor volumes of these 21 consecutive patients did not appear to have an even distribution, that most cases were clustered in the small range, and only two cases in the relatively high range. Differences between series may be relatively larger for high volume tumors than those of small volume tumors. More cases need to be collected to have a better sample of varying tumor volumes on the ICC results. Finally, we did not assess the perfusion by DCE-MRI. The correlation between parameters by those two perfusion modality would be an interesting topic for future investigation.

In conclusion, ASL is a promising non-invasive MRI technique that could assess NPC perfusion quantitatively. T2WI-FS/ASL fusion images could overcome the limitation of poor anatomy definition by ASL images alone and short of contrast between tumor to surrounding tissue by T2WI-FS alone, therefore had potential to increase the confidence of radiologist to evaluate the tumor extent, especially to delineate the radiotherapy target region for the patients who cannot receive contrast media administration.

## Materials and Methods

### Patients

This prospective study was approved by the institutional review board at Cancer Institute & Hospital, Chinese Academy of Medical Sciences. Informed consents were obtained from all participants. This study was carried out in accordance with the Declaration of Helsinki.

Within the period from March to June 2014, 22 consecutive patients with untreated NPC confirmed by pathology received MRI before nasopharyngoscope biopsy and treatment. Only one case was excluded in this population as this patient did not receive contrast injection due to allergic constitution. As a result, 21 cases were included in this study, and this population was comprised of 16 men (76.2%, 16/21) and 5 women (23.8%, 5/21) with a mean age of 48 years (range 21 to 75 years). The pathology showed undifferentiated type of non-keratinizing carcinoma in 11 cases (52.4%, 11/21) and differentiated type of non-keratinizing carcinoma in 10 cases (47.6%, 10/21). According to the seventh edition of the AJCC staging system^18^, there were 2 cases in stage T1 (9.5%, 2/21), 3 cases in stage T2 (14.3%, 3/21), 9 cases in stage T3 (42.9%, 9/21) and 7 cases in stage T4 (33.3%, 7/21).

### MR Scan

MR scans were performed on a 3.0 T whole body MR system (Discovery MR750, GE Medical Systems, Milwaukee, Wisconsin, USA) with an 8 channel head and neck phase array coil. Non-enhanced series included: axial T1WI (fast recovery fast spin echo, FRFSE, TR = 494 ms, TE = 13.63 ms); axial T2WI with IDEAL (FRFSE, TR = 4000 ms, TE = 85 ms, NEX = 1, bandwidth = 41.67 kHz, thickness = 5 mm, slice gap = 1 mm, FOV = 26 cm, matrix = 288 × 224, TA = 160 sec); axial DWI (diffusion weighted imaging, TR = 7075 ms, TE = 74 ms, b value = 800 s/mm^2^); sagittal T1WI (FRFSE, TR = 418 ms, TE = 12.35 ms). The arterial spin labeling sequence used was a 3D fast spin echo (FSE) spiral based pseudo-continuous pCASL sequence (NEX = 3, bandwidth = 41.67 kHz, thickness = 3 mm, slice gap = 0 mm, FOV = 24 cm, TE = 11.1 msec, PLD 1025 msec [1.0 sec]: TR/TA = 4326 msec/262 sec, PLD 1525 msec [1.5 sec]: TR/TA = 4569 msec/276 sec, PLD 2025 msec [2.0 sec]: TR/TA = 4781 msec/288 sec).

Contrast-enhanced scan was performed using 3D liver acquisition with volume acceleration-extended volume (LAVA-XV) after injection of Gadolinium-DTPA-BMA (Ominscan, GE lifeScience, China) with a dose of 0.2 ml/kg and rate of 2.0 ml/s. An initial axial dynamic contrast-enhanced MRI (DCE-MRI) was conducted over a scan duration of 240 s. Axial, sagittal, and coronal scan series were followed with a 3D fast spoiled gradient-recalled-echo, FSPGR. The parameters of the axial enhanced T1WI were TR = 295 ms, TE = 2.86 ms, NEX = 2, bandwidth = 31.25 kHz, thickness = 5 mm, slice gap = 1 mm, FOV = 26 cm, matrix = 320 × 256, TA = 120 sec. The spatial coverage of the axial T2WI-FS, ASL and enhanced T1WI were identical and it covered a range from frontal sinus to oropharynx in order to include all nasopharyngeal tumor, which allows appropriate fusion of the ASL and the T2WI/FS images.

### Data analysis

All patient information was anonymized and de-identified prior to data analysis. All data were processed using the Advantage Workstation (ADW 4.6 version, GE, US). Fusion step was performed by Integrated Registration software on workstation. All the acquired images (T2WI-FS, ASL) contain the geometrical information in the image header, the fusion image can be simply done by matching the geometrical information, both slice-wise or within plane. The fusion process was mostly automatic and the observers were not involved in the fusion process. Two observers with 20 years (D.L.) and 10 years (X.Y.) experience in MRI diagnosis were blinded to the patient’s information. For measuring the tumor volume, observers measured all the tumor regions slice by slice on T2WI-FS, T2WI-FS/ASL (with PLD of 1.0, 1.5 and 2.0 s) and enhanced T1WI (enhanced axial FSPGR) respectively, then the volume is calculated by the worksttion by summing all the areas of the delineated regions in all the slices. Measurements based on those five image series were conducted one month apart to minimize bias. The BF of NPC and lateral pterygoid muscle were simultaneously measured by the two observers.

### Statistical analysis

SPSS Statistics Version 18.0 was used for statistical analysis (v. 18.0, Chicago, IL). The comparison of volume measurements included: 1) the inter-series correlation and consistency, which was the comparison between the measurements made using enhanced T1WI and those made using T2WI-FS and the T2WI-FS/ASL fusion images respectively by same observer, 2) the inter-observers consistency, which was the comparison between the measurements by the two observers based on the same image series. Spearman correlation coefficients were performed to determine the correlation. The consistency was evaluated using the Bland Altman plots, which were made of the mean values against the difference values, with the 95% limits of agreement calculated as the mean difference plus or minus 1.96 times the standard deviation of the difference. The two-way model average measures Intra-class correlation (ICC) and within-subject coefficients of variation (wsCV)^19^,^20^. The wsCVs were calculated using the methods of Bland and Altman as published on http://www-users.york.ac.uk/~mb55/meas/cv.htm. Comparison of blood flow (BF) on ASL between NPC and lateral pterygoid muscle was conducted by paired samples t test. *P* value of less than 0.05 was considered to be statistically significant.

## Additional Information

**How to cite this article**: Lin, M. *et al.* Consistency of T2WI-FS/ASL fusion images in delineating the volume of nasopharyngeal carcinoma. *Sci. Rep.* **5**, 18431; doi: 10.1038/srep18431 (2015).

## Figures and Tables

**Table 1:** Correlation of inter-series.

	Observer 1			Observer 2		
	Mean of tumor volume	r	*P*	Mean of tumor volume	r	*P*
T2WI-FS	15.77 ± 19.72	0.910	<0.001	15.47 ± 16.11	0.896	<0.001
T2WI-FS/ASL 1.0	17.52 ± 21.86	0.973	<0.001	18.72 ± 23.61	0.969	<0.001
T2WI-FS/ASL 1.5	17.15 ± 19.66	0.947	<0.001	19.38 ± 24.46	0.936	<0.001
T2WI-FS/ASL 2.0	17.10 ± 20.13	0.953	<0.001	18.26 ± 22.36	0.921	<0.001
Enhanced T1WI	18.04 ± 23.76	—	—	17.86 ± 23.80	—	—

The correlations between measurements made using four images series to those based on enhanced T1WI respectively.

**Table 2:** ICC of inter-series.

	Observer 1			Observer 2		
	ICC value	95% confidence interval	wsCV (%)	ICC value	95% confidence interval	wsCV (%)
T2WI-FS	0.978	0.945 ~ 0.991	21.61	0.951	0.878 ~ 0.980	24.21
T2WI-FS/ASL 1.0	0.997	0.992 ~ 0.999	14.76	0.988	0.969 ~ 0.995	18.00
T2WI-FS/ASL 1.5	0.984	0.926 ~ 0.994	18.11	0.984	0.961 ~ 0.994	22.07
T2WI-FS/ASL 2.0	0.989	0.972 ~ 0.995	18.65	0.980	0.950 ~ 0.992	22.96

The ICCs between measurements made using the four series respectively to those based on enhanced T1WI. ICC is the abbreviation of intra-class correlation. wsCV is the abbreviation of within-subject coefficients of variation.

**Table 3:** ICC of inter-observer.

	Observer 1 VS. Observer 2		
	ICC value	95% confidence interval	wsCV (%)
T2WI-FS	0.987	0.969 ~ 0.995	15.13
T2WI-FS/ASL 1.0	0.992	0.981 ~ 0.997	16.52
T2WI-FS/ASL 1.5	0.981	0.953 ~ 0.992	16.37
T2WI-FS/ASL 2.0	0.985	0.962 ~ 0.994	17.91
Enhanced T1WI	0.996	0.989 ~ 0.998	9.95

The ICCs were between measurements of the two observers based on the same image series.

**Table 4:** Comparison of BF.

	ASL1.0		ASL1.5		ASL2.0	
	NPC	LPM	NPC	LPM	NPC	LPM
BF (mL/min/100 g)	74.67 ± 18.05	15.01 ± 2.89	81.19 ± 19.25	20.04 ± 3.57	82.52 ± 21.59	21.15 ± 3.74
P	<0.001		<0.001		<0.001	
t	14.481		14.899		13.205	

BF is the abbreviation of blood flow. LPM is the abbreviation of lateral pterygoid muscle.
